# Perceptions of Food Hypersensitivity Expertise on Social Media: Qualitative Study

**DOI:** 10.2196/10812

**Published:** 2019-06-28

**Authors:** Richard James Thomas Hamshaw, Julie Barnett, Jeff Gavin, Jane S Lucas

**Affiliations:** 1 Department of Psychology University of Bath Bath United Kingdom; 2 Clinical & Experimental Sciences Faculty of Medicine University of Southampton Southampton United Kingdom

**Keywords:** social media, food allergy, food hypersensitivity, celiac disease, food intolerance, interviews as topic, qualitative methods

## Abstract

**Background:**

Seeking and sharing information are the primary uses of the internet and social media. It is therefore vital to understand the processes individuals go through when engaging with information on these diverse platforms, especially in areas such as health- and risk-related information. One important element of such engagement is evaluating and attributing expertise to others.

**Objective:**

This study aimed to explore how meanings around expertise in relation to food allergy and intolerance (food hypersensitivity) were constructed by 2 groups of social media users: (1) those who use platforms for reasons relating to food hypersensitivity and (2) those seen as experts by this community.

**Methods:**

Survey participants were asked open-ended questions to identify potential experts in food hypersensitivity issues on social media and to discuss their reasoning for their choices (n=143). Subsequently, 8 adult social media users with experience of managing food hypersensitivity and 5 participants designated as experts by those users took part in email interviews. Survey and interview data were analyzed thematically using Braun and Clarke’s approach.

**Results:**

Judging expertise on social media is a complex and multifaceted process. Users might be judged as experts through their professional background or their experience living with food hypersensitivities. How users behave on social media and the traces of their Web-based activity can influence how others will see them. Such considerations are both measured and moderated through the social media community itself. Findings highlighted how social media often act as a supportive information tool following a diagnosis, but this also raised concerns regarding the scenario of patients not being able to access suitable vetted information.

**Conclusions:**

This work has implications for understanding how users perceive expertise on social media in relation to a health concern and how information assessments are made during the management of risks. Findings provide practical insights to both medical and organizational stakeholders involved in the support of those living with life-changing conditions, such as food hypersensitivities.

## Introduction

### Background

In today’s digital age, people attend to the information they encounter on social media; seeking and sharing health-related information is a common practice [1-3]. However, in situations where there is the possibility of negative health consequences, it is important that people are acting on accurate and reliable information. Judgments about the expertise of the source are an important part of this, and it is, therefore, important to know what the heuristics for judging expertise are in the context of social media [4]. One such situation with potential negative consequences to health is food hypersensitivity—conditions associated with the need to avoid specific foods that cause adverse reactions [5]. By gaining an insight into perceptions of expertise in food hypersensitivity on social media and from the perspective of those living with hypersensitivity and those deemed to be experts in this area, we can further shed light on the dynamics of expertise on social media in relation to food, health, and risk. A greater understanding of the factors that affect an individual’s perceptions of expertise online may have implications for agencies and organizations that support people with health concerns.

### Food Hypersensitivity

Food hypersensitivity occurs in people who experience reproducible adverse symptoms when consuming specific foods and denotes both food allergy and nonallergic food hypersensitivity, for example, food intolerance and celiac disease [6]. Living with food hypersensitivities involves constant risk assessments surrounding the food one consumes. This is especially the case when eating outside the home [5,7-9]. Those with food intolerance wish to avoid repeatable adverse reactions to food, such as bloating, constipation, vomiting, and diarrhea. Celiac disease is an autoimmune disease caused by the immune system reacting to the protein gluten (found in the cereals wheat, barley, and rye), which leads to similar adverse reactions, but it can have long-term consequences if undiagnosed, such as anemia, fatigue, and weight loss [10]. Individuals who are allergic to certain food items must avoid consuming allergens that could lead to a reaction called anaphylaxis (associated with breathing difficulties, sudden drop in blood pressure, and which may be fatal). Given these characteristics of food hypersensitivity, this is an ideal domain within which to explore attributions of expertise on social media because misinformation may have significant consequences [11,12]. The aim of this study was to explore how social media users and perceived experts in food hypersensitivities on social media construct meanings around expertise. To this end, we will first consider how expertise can be defined and interpreted, how internet users seek information on social media, the cues they use to assess potential expertise, and how they validate the information they encounter.

### Defining Expertise

When attempting to define expertise, experts typically have comprehensive and authoritative knowledge in a specific area [13]. They are well regarded by their peers, relay accurate and reliable information, and have gained extensive knowledge through their experience [14]. Being an expert is normally considered a good thing to be respected or cited in relation to one’s area of expertise [15]. Expertise is largely an attribution; someone is usually considered an expert because others see them as experts [15]. Expertise typically encompasses assessments of credibility, trustworthiness, believability, and accuracy of information [16]. Expertise might be assessed through academic qualifications, years spent in a specific role, or experience [17]. The importance of experience, however, highlights how distinctions between experts and lay persons can be flexible and dynamic, for example, if a lay person has experience in a certain area [18]. Expertise is contextually valuable; an individual may know a lot about specific contexts and situations (eg, from their life experiences) but little outside of that environment [18]. Whether expertise on specific social media platforms holds true for expertise in other contexts (eg, offline or through different platforms) is worth consideration; Sternberg and Frensch [15] note “experts in one place or one time are not necessarily considered to be experts in another place or another time.”

### Seeking Information from Experts on Social Media

Seeking and sharing information are primary uses of the internet and social media [12,19-21]. In comparison with more traditional media, social media allow users to communicate in a reciprocal way, exchanging knowledge, sharing opinions, or challenging information from others [22]. Social media encompass a variety of internet-based platforms that use the technological advances of Web 2.0, associated with collaboration and user-generated content [23]. Some social media sites are designed explicitly to enable and encourage interactions, such as Facebook and Twitter. Others embody some functions of social media (eg, posting, commenting, liking, and sharing) but where this is not the primary purpose of the site, such as forums, chat rooms, and comments systems following Web-based news articles or published media (eg, blogs). Health information seekers can readily connect with those who share similar health concerns [24,25]. In fact, information circulated among peers, especially those perceived to be similar, may be more influential than formal expertise [25,26]. Social media can offer access to other people living in similar circumstances, and as a result, those managing health conditions often turn to their social media peers for help, perhaps for emotional peer support, for example, from other parents of allergic children [8,27]. This instant and supplementary access to other perspectives contrasts with information provision practices within a more formal medical setting.

Web-based information-seeking practices can be dependent on individual characteristics or motivations of the user. Metzger and Flanagin [16] highlight how the level of accuracy an information seeker is aiming for, their *accuracy goal* [28], will vary when using the internet. When using social media, for example, information seeking might be quite casual, where accuracy in the information is less crucial (eg, searching for ideas on Pinterest), or purposeful, where getting the correct information is important (eg, around a medical concern). Information in line with current beliefs tends to be noticed and valued more, with discrepant information more likely to be disregarded, even when opposing arguments are well argued and evidenced [29].

Thriving groups of users with specific health concerns exist on social media, for example, users with diabetes on Facebook [1] and food allergic and intolerant individuals on Twitter [30]. Those with health concerns are sharing experiences as well as gaining independence and self-sufficiency through the internet [3]. For people caring for loved ones, social networking platforms and forums comprising people in similar circumstances can be a source of reassurance and support [24]. However, having many *authors* of relevant information on social media can pose difficulties for credibility assessments because the origin and development of a source can become difficult to authenticate [16]. A lack of verification systems or formal gatekeepers and the fact that, in most cases, any user can publish or post information on the internet mean that it is important to understand how people assess the credibility of the information they encounter [2,16,20,31]. In light of this, we now turn to consider the cues used to assess information on the internet. Metzger and Flanagin [16] provide a useful framework for considering the kinds of cues that could affect perceptions of expertise in terms of source, author, and message assessments.

### Source Assessments

In internet research to date, source has often referred to the websites that present information; *source* and *site* are often used interchangeably. Cues to credibility provided by the source of information have included the following: design, navigability, absence of errors, links to other reputable sources (or academic citations), evidence of sponsors, or whether the site makes money from advertising or product promotion [16,32-34]. In a review of several studies about Web-based health information seeking, Cheever and Rokkum [35] highlight how testimonials or comments from other users on Web content are increasingly being used to assess the credibility and veracity of Web-based content. However, in the realm of social media, a *source* is more challenging to define. It might refer to the platform user profiles are held on (eg, Facebook, Twitter, or Instagram), but user profiles themselves might be seen as separate sources, as they hold much of the information to be considered a site in their own right (eg, their own Web address, content, and layout). Research around website assessments of credibility are likely to relate to certain sources such as blogs, but the multidimensional nature of social media does not translate so easily: a platform that might be considered credible by users may not necessarily always contain credible sources of information although familiarity with a specific platform may give a user better tools to make assessments about the information or users within [36,37].

### Author Assessments

Certain characteristics of the authors of Web-based material can help other users assess the expertise of the published information. Metzger and Flanagin [16] highlight factors such as the author’s qualifications, reputation or professional association, available contact information, and lack of commercial motives. Social media allow us to make quite detailed judgments about individuals we encounter, as users leave traces of their Web-based activity. For example, having many followers on one’s social media accounts or having forum answers ranked highly by other members could be potential signals of expertise [38]. Similarly, the reactions of others may have some bearing on how individuals judge the expertise and reputation of social media accounts; shares, retweets, comments, and likes can be used as indicators to affirm how others see sources of information on the internet [38,39].

Often in the absence of an official or qualified source, users with experiential knowledge or *situated understandings* may be mobilized to offer additional insights on an issue [40]. People with long-term illnesses may become expert in their particular condition based on experience and specific contexts that relate to their health concern [18]. Cues relating to shared and lived experience can lead to a sense of collective trust. For example, parents of children with a newly diagnosed food allergy were seen drawing on the expertise of other parents they knew had gone through the same sorts of issues [27]. In another example, users of a multiple sclerosis (MS) support forum were seen to share experiences and treatments in addition to (what was considered) static Web-based advice monitored by the professional MS bodies [41].

There are several cues that may be used to infer the credibility of the Web-based content: clarity of writing, accuracy, presence of bias, recency of information, and supporting evidence [16,32-34]. In the realm of Web-based health information, the use of medical discourse holds high social status and legitimacy [42], increases a user’s social credibility, and is often a cue to expertise. Furthermore, using community terminology (such as abbreviations and acronyms) as well as presenting information as factual are also ways of performing expertise [43-45]. Cues of a social nature that are attached to social media posts, such as comments, likes, and shares, are likely to play a significant role in how users make message assessments, for example, whether they accept or trust the information provided or wish to participate in the discussion themselves [35,39,46].

### Objectives

In this study, we investigate how users construct meanings around expertise on social media in the area of food hypersensitivity. We explore the construction of expertise from 2 user perspectives: (1) social media users who are food hypersensitive (FH) or parents of FH children and (2) perceived experts in food hypersensitivities within the FH social media community.

## Methods

### Design

Initial exploratory data were obtained from open-ended qualitative questions on a Web-based survey relating to social media use for FH concerns [12]. These data were combined with subsequent in-depth qualitative email interviews. In total, 13 email interviews were conducted with 8 FH adults and parents of FH children who use social media (hereafter referred to as FH participants) and 5 perceived experts in food hypersensitivity on social media. Given the focus of the study, we knew participants were confident to engage online; social media users are likely to be technologically able, and access to the internet would not be an issue. Email interview techniques were chosen here, as they are particularly appropriate when participants are asked about something that they are unlikely to have explicitly considered before [47,48]. The approach gives participants time to contemplate questions. We were able to explicitly ask participants to consider their responses before replying, as well as provide examples from their own social media activities if it helped them get their point across or jog their memory. The ability to review responses sets this approach apart from many other forms of qualitative data collection and can provide more articulate responses and richer, more focused data [48]. This study was exploratory in nature, and to avoid restricting the narrative of interview discussion, social media were considered in a broad sense. This allowed participants to discuss perceptions relating to their understanding of their own social media use. Thus, participants could refer to various types of social media, as outlined in the introduction, such as well-known platforms themselves (eg, Twitter, Facebook, and Instagram) or chatroom websites and online support groups.

### Participants

From the survey, 143 participants completed 2 open-ended questions. Table 1 shows the demographic details and social media use descriptives relating to this sample. Overall, 2 groups of interview participants were recruited. One consisted of FH social media users who identified potential experts in food hypersensitivity within their social media networks. This sample of users was recruited from the survey and had given permission to be recontacted for this follow-up study; these participants are not included in the 143 participants mentioned previously [12]. Table 2 shows the demographic characteristics of interview participants. Another sample comprised users identified by the FH participants as experts in relation to food hypersensitivity. From respondents on the previous FH survey, 98 potential experts were identified; this list contained multiple duplicates, and following inclusion criteria for accounts managed by individuals (as opposed to larger organizations) and those contactable through social media or public email addresses, a list of 30 potential experts was created. From this list, 16 users were randomly selected and invited to participate; 5 took part. The professions and backgrounds of experts varied, comprising a health journalist and writer, food policy official, FH travel writer, social media discussion group moderator, and FH recipe blogger. There were 4 female experts and 1 male expert. The data collection period for the survey was January to March 2017, and for the email interviews, April to May 2017.

**Table 1:** Demographic characteristics for survey participants.

Descriptive		n (%)
Female		131 (91.61)
Male		10 (6.99)
Preferred not to say		2 (1.40)
**Age (years)**		
	18-24	9 (6.29)
	25-34	36 (25.18)
	35-44	44 (30.77)
	45-54	31 (21.68)
	55+	23 (16.08)
**Education level**		
	University degree	85 (59.44)
	Commercial/technical diploma	32 (22.38)
	Secondary education	22 (15.38)
	Prefer not to say	4 (2.80)
FH^a^ adults		92 (64.34)
Parents of FH children		51 (35.66)
**Diagnosis^b^**		
	Allergy	64 (44.76)
	Celiac disease	59 (41.26)
	Intolerance	86 (60.14)
**Speed of reaction**		
	Immediate	47 (32.87)
	From 1 hour	36 (25.17)
	1-24 hours	47 (32.87)
	>24 hours	13 (9.09)
**Reaction causing allergen^c^**		
	Peanuts	39 (27.27)
	Nuts	28 (19.58)
	Cow’s milk	48 (33.57)
	Gluten	81 (56.64)
	Eggs	29 (20.28)
	Fish	4 (2.80)
	Crustaceans	7 (4.90)
	Molluscs	7 (4.90)
	Soya	18 (12.59)
	Celery	1 (0.70)
	Mustard	4 (2.80)
	Lupin	3 (2.10)
	Sesame	14 (9.79)
	Sulfur dioxide	5 (3.50)
	Other	27 (18.88)
**Social media use**		
	Facebook	135 (94.41)
	Twitter	73 (51.05)
	Instagram	50 (34.97)
	Pinterest	56 (39.16)
	Snapchat	21 (14.69)
	YouTube	73 (51.05)
	TripAdvisor	63 (44.06)
	Tumblr	6 (4.20)
	Support groups	71 (49.65)
	Comment forums	33 (23.08)
	Other	4 (2.80)
**Frequency of use**		
	<Less than 1 hour	33 (23.08)
	1 hour (approximately)	50 (34.97)
	2 hours (approximately)	31 (21.68)
	3 hours (approximately)	10 (6.99)
	4 hours (approximately)	3 (2.10)
	>4 hours	16 (11.19)

^a^FH: food hypersensitive.

^b^Individual may have more than 1 type of diagnosis.

^c^Individual may experience reactions from more than 1 allergen.

**Table 2:** Demographic characteristics for food hypersensitive interview participants.

Descriptive		n (%)
Female		7 (87.50)
Male		1 (12.50)
**Age (years)**		
	18-24	0 (0)
	25-34	3 (37.50)
	35-44	4 (50.00)
	45-54	0 (0)
	55+	1 (12.50)
**Education level**		
	University degree	7 (87.50)
	Commercial/technical diploma	1 (12.50)
FH^a^ adults		4 (50.00)
Parents of FH children		4 (50.00)
**Diagnosis^b^**		
	Allergy	5 (62.50)
	Celiac disease	3 (37.50)
**Speed of reaction**		
	Immediate	5 (62.50)
	From 1 hour	2 (25.00)
	1-24 hours	1 (12.50)
	24 hours +	0 (0)
**Reaction causing allergen^c^**		
	Cow’s milk	2 (25.00)
	Nuts	3 (37.50)
	Eggs	1 (12.50)
	Gluten	3 (37.50)
	Peanuts	1 (12.50)
**Social media use**		
	Facebook	8 (100)
	Twitter	5 (62.50)
	Instagram	3 (37.50)
	Pinterest	3 (37.50)
	Snapchat	1 (12.50)
	YouTube	5 (62.50)
	TripAdvisor	4 (50.00)
	Tumblr	0 (0)
	Support groups	5 (62.50)
	Comment forums	4 (50.00)
	Other	0 (0)
**Frequency of use**		
	<1 hour	2 (25.00)
	1 hour (approximately)	4 (50.00)
	2 hours (approximately)	2 (25.00)

^a^FH: food hypersensitive.

^b^Individual may have more than 1 type of diagnosis.

^c^Individual may experience reactions from more than 1 allergen.

### Materials

The open-ended questions from the larger FH social media use survey that formed part of this analysis asked participants to identify any social media accounts they considered expert in FH issues and discuss their reasoning for recognizing these sources as experts [12]. The subsequent email interview schedules covered questions relating to typical use of social media and aspects of accounts that may be considered as cues to expertise (Multimedia Appendix 1). Questions were informed by the literature and were checked and clarified with the research team and other colleagues to minimize the possibility that participants would require clarification or explanation, which would have unnecessarily increased the number of email exchanges. FH participants were asked questions around their reasons for highlighting specific users as experts. Questions to experts asked participants to reflect on their own expertise and their thoughts on being perceived as an expert by other users. The schedules were intended as guides to the interview structure with a degree of question flexibility for follow-ups on relevant information. Separate email invitations and consent forms were developed for each group.

### Procedure

As mentioned previously, open-ended responses were included from a previous survey study; these asked participants to consider potential experts in FH issues on social media and provide some reasons for their choices. Participants from this survey study, who agreed to take part in the email interviews and provided informed consent, were emailed the first set of interview questions. Similar to face-to-face interviews, subsequent questions followed up on the aspects of previous responses and asked for elaboration or further explanation, as well as providing the next schedule questions. On completion, a final debriefing email was sent to thank participants for taking part and to give further information about the study. Due to the longer duration of email interviews, a £20 Amazon voucher (equivalent to US $25.33, Can $ 33.62, AUS $36.39) was given to interviewees as compensation for their time and to thank them for participating. On average, there were 5 email exchanges (ie, email sent and responded to) with each participant, a minimum of 3 and maximum of 7. Typically, each interview email included 2 or 3 questions (with probes) for participants to respond. Email interactions were anonymized and saved as Microsoft Word documents to facilitate analysis. Pseudonyms replaced names of individuals referred to in the interviews. Names of organizations were retained. Participants were able to use their preferred internet-enabled device to respond at a time and place that suited them.

### Ethics

Email interview participants were asked to give consent by typing their name and date in the final section of the email information sheet to confirm they understood the study information. An email interview approach itself can resolve some ethical considerations associated with typical face-to-face interviews; participants must actively click to send responses, and this arguably acts as a second phase of consent—the risk of participants inadvertently sharing something is much lower. Data security and confidentiality remained paramount. Data were stored on secure password-protected university servers, and names or associations linked to participants were removed from transcripts. Approval to contact participants from the previous survey study was granted by the University of Bath ethics committee (reference: 16-146), and approval for this project was also granted by the same committee (reference: 17-004).

### Analysis

An in-depth qualitative thematic analysis was conducted, following the guidelines set out by Braun and Clarke [49,50]. Early stages of analysis featured thorough familiarization with dataset content and development of initial codes (eg, through annotation of interesting elements relevant to the research questions). Following initial descriptive first-order coding, codes were grouped into more specific second-order codes, which were used to develop overall themes. Final themes were reviewed and refined to ensure that they appropriately explained their content and considered as much of the data as possible. The number of interviews analyzed would be considered appropriate in line with typical email interview samples of 5 or more participants [51]. Guest et al [52] note, when coding for overarching themes, a sample of 6 interviews can be sufficient to enable the development of meaningful themes and beneficial interpretations. However, the addition of 143 shorter but detailed open-ended answers to questions relating to the reasoning behind judgments of expertise for FH social media sources complemented this more in-depth sample. Furthermore, the homogeneity of our sample (the FH concerned) and clear aims surrounding perceptions (of expertise) further support the suitability of our sample size [52].

## Results

### Thematic Analysis

In outlining findings, we discuss observations across and within groups to develop a clear narrative that highlights associations and overarching concepts relating to perceptions of expertise in food hypersensitivity on social media. In quoting from interview participants and survey respondents, FH demographic information is also highlighted: FH *Adult* or *Parent* of an FH child; sensitivity as *Allergy*, *Celiac*, or *Intolerance*. Interviewees are denoted by an *I*, and survey respondents with an *S*, both followed by their participant number. Perceived expert interviewees are represented by an *E* and participant number. Overall, 4 main themes were identified in the data: (1) discerning traditional expertise on social media, (2) expertise acquired through lived experience, (3) cues to expertise in social media content, and (4) cues to expertise afforded by social media practices.

### Discerning Traditional Expertise on Social Media

There was clear recognition across the data that 1 marker of expertise on social media was a qualified professional (often a medical professional). Participants highlighted how using an official title (eg, dietitian) or job description on the internet increased the likelihood of attributions of expertise. Claims of qualifications were similarly unproblematically equated with having expertise. Those working for or associated with experts within the field were also considered more credible, as well as links to research outputs:

To consider them an expert they would either be working within the field of allergy or involved in research.

I4-Parent-Allergy

Dietician is a protected title in the UK.

S74-Adult-Celiac

Published academic, so I would consider trustworthy.

S9-Adult-Intolerance

Participants often noted how they had met perceived experts in an offline capacity (eg, at conferences or events) or that an expert was, in fact, their own or their child’s doctor or nurse:

I know some of the doctors quoted from our time at the allergy clinic.

S37-Parent-Allergy

I follow immunologists or doctors I have heard about from Anaphylaxis Campaign or Allergy UK.

S119-Parent-Allergy

Many of the judgments of expertise here are based on attributes outside the realms of social media. Here, expertise is not extrapolated from what the perceived expert user is *doing* online, but rather from the markers of expertise associated with them such as qualifications, publications, and external relationships. Social media are not an influencing factor in their possession of expertise, which would also presumably exist as a perception through patient and peer assessments in the offline world. In contrast, without the influence of a social media network, one might assume the expertise of nonqualified individuals discussing FH issues (as will be discussed in our next theme) would not stretch much farther than their personal, physical networks.

Traditional sources of expertise formed a benchmark against which users sought to discern the credibility of social media information. Social media information was scrutinized by comparison with traditional materials more likely to have been checked and evidenced with scientific backing or recommended by qualified health professionals:

Social media gives a platform to people who can say almost anything they like. When I was first diagnosed...I noticed there were a lot of contradictory information. As I was given an information pack by the NHS I used this as level 1 point of reference and compared what I found on the internet to this so I could sort the facts from the hearsay.

I6-Adult-Celiac

Furthermore, and as I6 highlights previously, receiving a new diagnosis heightens uncertainty and concern about the trustworthiness of information needed to manage hypersensitivities on a daily basis:

There are too many groups that people use as a platform for personal preferences, views and experiences. It can be daunting for somebody newly diagnosed to know what is what.

S7-Parent-Allergy

I think people new to the world of allergy struggle to see what is correct and what isn't.

I8-Adult-Allergy

However, it was often the perceived shortcomings in the support from qualified professionals that led FH individuals to seek support elsewhere:

However, my personal experience...is you get your diagnosis, you go away with your list of foods and your left to it. Yes you have a follow up appointment with the dietician 6 months after and can call for advice. But I feel you are just left to work the rest out.

I1-Parent-Allergy

Those coming fresh to social media looking for answers after getting short shrift from their GP etc. are more likely to fall into the trap laid by self-styled experts.

E2

The recently diagnosed patient sought information that could be trusted at a time of vulnerability and uncertainty. The acknowledgment of professional titles, qualifications, and experience with experts outside the realms of social media were key markers of expertise online. Thus, the locations and boundaries of expertise begin to be defined: signifiers of traditional medical expertise were valued around the process of diagnosis.

### Expertise Acquired Through Lived Experience

The concept of expertise developing through experience featured strongly for both FH participants and perceived experts. Having lived (or cared for someone) with food hypersensitivity conveyed expertise in managing the condition:

Having easy access to people who have already been through it who share this knowledge may mean people are seen to be “expert” sources of information

I5-Adult-Celiac

The nature of expertise that living with FH conveys is associated with day-to-day living, for example, managing a child’s allergy at school, appropriate places to eat out, or guidance on eating out in other countries. Such postdiagnosis day-to-day expertise is not seen as being available from the medical community but rather from those whose expertise stems from their personal encounters with the issue. These experts can be accessed through social media:

You can’t get more expert than someone who appreciates and lives with the strains, stresses, worries of an allergy; and I feel that Facebook support groups provide this. Medical professionals know the “medical” bit but they don’t deal with the day to day living.

I1-Parent-Allergy

The role of charities seemed to occupy a middle ground in terms of the expertise attributed to them. The provision of good advice was valued but could be associated with a lack of emotional resonance though being run by those who had personal experience of FH accorded greater credibility:

Gov and charities are a good source but their content lacks emotion and passion.

S52-Adult-Celiac

They’re not run by professionals but others in similar situations and have been through the diagnosis etc!!! Often more helpful than the medical profession and I belong to the medical profession.

S85-Parent-Allergy

Although perceived benefits of information from those with experience was clear, there was also an appreciation that the information provided was a function of differences in the ways FH individuals approach their condition or differences in their conditions (eg, reaction severity or types of allergy or intolerance). Those who have lived with a food hypersensitivity for several years and feel confident in their lack of reaction, for instance, may take certain consumption risks:

Variations in the way some people may take “risks” could create some confusion particularly to those who have just been diagnosed.

I5-Adult-Celiac

Having said that, there was not an unthinking or automatic acceptance of the advice given by those deemed as experts by experience. There was some acknowledgment that the experience of different reactions to the same allergen (eg, cow’s milk allergy vs lactose intolerance) could be associated with information that may be inappropriate. Therefore, it was not food hypersensitivity, in general, that conveyed expertise but rather it was having the “same condition” (S140-Adult-Celiac) and the “same intolerances” (S28-Parent-Allergy). This demarcation of expertise was also evident from the perspective of the experts themselves. One such participant, an experienced FH mother, outlined the boundaries of her expertise explaining that she would avoid handing-out health advice and rather point people in the direction of medical professionals:

I share my own experiences but never give medical advice—I always refer to a doctor or official resource...I would say I am an “expert” parent in the sense that I have experience managing allergies day-to-day, and can advise on issues such as handling school and nursery.

E2

The online FH community were accorded a role in helping to moderate the kinds of information being shared and ensure that it was appropriate and credible. For example, a weekly Twitter discussion hour around allergy matters, #AllergyHour, was 1 location where this occurred:

I often join in with...#allergyhour where you can ask anything allergy-related and someone will have experience to share. There is a tremendous support network on Twitter. We very much see ourselves as an allergy family.

E4

Information would be subject to a process of social validation. By asking other individuals experienced in managing FH concerns their opinion on specific matters or by others sharing the knowledge they had gained from expert sources elsewhere (eg, in managing children’s allergies, eating out, recipes, or recommendations for medical advice or treatment), communities became a trusted site of knowledge. Members of these social media communities were more confident to use information or attribute expertise if it had been vetted or accepted by other trusted users:

They are other parents in the same boat sharing information which they have either learned themselves or sharing information from doctors, health visitors, dieticians.

S109-Parent-Allergy

Thus, there were 2 pillars that buttressed those seeking credible information on social media: (1) information about the experience of living with food hypersensitivity and (2) medical information. Social media support postdiagnosis was viewed as legitimately sought and provided in relation to the experience of living with food hypersensitivity, although it was recognized by some at least that this may be inappropriate because experiences of food hypersensitivity varied greatly. Those who had been accorded as experts on social media were reticent to give medical information and noted the pitfalls of doing so.

### Cues to Expertise From Content

The first 2 themes have primarily considered attributions of expertise located in the characteristics of that person: their qualifications or experience. However, the nature of the information being posted or available on social media was also a marker of potential expertise. For example, the relevance and novelty of social media communications served as cues to expert status:

I don’t tend to share material or news which is already “doing the rounds” or has been shared widely already by others—I’ll trust that my followers will already have seen it.

E1

Posts relating to current issues. They give out useful information that is updated.

S100-Adult-Allergy

Expert information needed to be factually correct, and this was signified by links to research journals, official publications, or trusted sources:

I share from credible sources, but in all cases I read the article or link on the Tweet to make sure I am reposting something which is accurate, share interesting materials/facts/research.

E3

If someone had credible information backed up with scientific research that was a) new to me and b) working, that would be great.

S51-Adult-Celiac

Information is based in fact and scientific evidence.

S34-Parent-Allergy

In contrast, those who did not give evidence for their claims or were promoting information users felt had no medical or research backing were not viewed as credible:

In what way might you consider someone on social media as non-expert?

Researcher

People who claim they cured their allergy with simple lifestyle changes such as buying a salt lamp. Or people pedalling Vega tests which have no medical backing whatsoever.

I4-Parent-Allergy

People who don’t know what IgE mediated allergy is or do not know the difference between lactose intolerance or CMPA. People who think someone with CMPA can have a little dairy and be okay.

I3-Parent-Allergy

Some did not expand on the grounds of how they would make their judgment but simply stated the nature of the content that they would attend to: it needs to be *accurate*, *relevant*, and *evidenced*.

User profile information was also seen to contain certain markers that users may use to assist them in assessments about the information presented there. One such example was the number of followers:

People like the collectiveness. They, subconsciously perhaps, believe if lots of other people are following/believing someone there is safety in numbers and it must be true.

I3-Parent-Allergy

I think they became to be seen as an expert by blogging originally and then creating the website and Facebook group. This has then attracted a large number of followers and so then people consider it as expert/knowledgeable simply because of the number of followers and it becomes self-fulfilling.

I3-Parent-Allergy

However, others took the opposite view, seeing follower numbers as a warning sign rather than a sign of expertise:

I fear a lot of people equate lots of followers with knowledge or expertise.

E4

There is definitely fake authority imbued by someone who has tens of thousands of followers—for instance some celebrities or self-styled food gurus. Social media makes it easier for these people to have a voice.

E2

Thus, the number of followers associated with content may be seen as a potential heuristic for assessing the accuracy and relevance of information; the following may itself act as a form of evidence. However, there are concerns if this following is not indicative of more traditional expertise or clear lived experience.

### Cues to Expertise Afforded by Social Media Practices

In addition to considerations around the nature of the information available on platforms, participants noted how the way users behaved or performed on social media may be a marker to expertise. Some participants noted that the degree of interaction in social media engagement was pertinent to judgments about expertise:

I look for accounts that interact with other people...I don’t have a lot of time for accounts that only retweet other people’s tweets

E4

I think the perception is due to the fact I respond to tweets, correct factual errors, I am quite vocal.

E3

The practice of deferring to other users considered to have more expertise in an area and being open to feedback were also seen as markers of expertise:

I also look for non-qualified people who defer to qualified people—always a good sign.

E1

The two bloggers I referred to in my previous responses tend to offer advice and welcome feedback rather than making statements they believe to be fact.

I6-Adult-Celiac

The option on social media platforms to tag other users within posts and thus draw them into discussion supported these engaged interaction practices, and such exchanges are readily visible in the history of related posts or feeds. Similarly, evidence of connections with key FH stakeholders served to warrant credibility or expertise. These connections included relationships with associated charities, businesses, or organizations:

[The Facebook group moderator] talks directly to companies and gains assurances that certain products are completely nut free. This has led to the Facebook group being very popular as lots of people value [their] knowledge and the contacts [they] have.

I2-Adult-Allergy

I follow immunologists or doctors I have heard about from anaphylaxis campaign or allergy UK.

S119-Parent-Allergy

A mark of trusted expertise was brokering the content supplied by relevant external stakeholders, or to put this another way, the credibility of the content was enhanced when it was mediated by a trusted expert. Conversely, some noted that people may attempt to align themselves with the official profiles of organizations to project a greater sense of legitimacy. The ability to include and link to others on social media affords users who may not have expertise the possibility of enhancing their presentation of authenticity:

The individuals have yet to demonstrate themselves in the arena, the audience have yet to form a view on whether they are credible and borrowing from the reputation of others can ease this.

E3

In short, features such as liking, sharing, replying, and tagging on social media sites provide ways that users can observe the behaviors, attitudes, reputations, and level of engagement of accounts they wish to make assessments about. At the same time, FH participants noted that such features can assist in less authentic users attempting to portray a certain level of credibility or expertise within social media networks. This final point highlights how the affordances of social media platforms can, on the one hand, hinder the efforts of FH users seeking support online, but, at the same time, may support the FH community in moderating inappropriate information, for example, through calling out, tagging, or publicly warning others about posts.

## Discussion

### Principal Findings

In exploring how those managing food hypersensitivity and perceived experts constructed meanings around expertise, we identified 4 themes. The first and second themes were associated with the location of expertise: either being valued as an expert in a more traditional manner (eg, through qualifications and professional knowledge) or acquired through experience managing food hypersensitivity. Both forms of expertise were valued, and traditional expertise was most often unchallenged and taken for granted. The third theme highlighted the specific cues to expertise in social media content. Users were seen to attend to various markers of expertise in the FH realm, such as evidenced, accurate, and relevant posts. The final theme considered the effect of social media practices or behaviors. Expertise may be assigned if a user is seen to engage with the FH social media community as well as draw on and interact with other potential expert users.

A key cross-cutting issue related to the concerns felt around the time of diagnosis for FH individuals and parents. Several participants across both FH participants and experts emphasized the importance of patients being able to get access to correct information, and this was not always guaranteed when using social media. It is a paradox that social media provide important perspectives postdiagnosis about managing the condition—perspectives that are not available through traditional medical channels often instrumental in diagnosis—and yet they cannot be unproblematically taken on board—cues to expertise have to be found and interpreted. A medical background or qualification was a taken-for-granted factor in defining expertise in the FH area. However, in the absence of expert knowledge, we see experienced FH patients and carers offering advice through social media about the day-to-day management of avoiding allergens. Research looking into internet use in patient-practitioner relationships has suggested that it would be beneficial for both parties if physicians used their knowledge to guide patients to approved sources [53], and this may help reduce anxieties surrounding users taking advice that may not correspond with medical opinion [54].

Social media were seen as providing a *treasure trove* of nonprofessional expertise [41] and highlighted the value placed on experiential knowledge or *situated understandings* [40]. However, participants were often clear to stress that they would frequently take information read on social media and consider it in line with more official (eg, National Health Service) materials and their own knowledge. It is not simply the case that social media information is considered as credible as more traditional media sources [16], it sometimes was used as a source on top of, and to complement, traditional materials. This finding has also been seen in parents of children recently diagnosed with food allergy; parents used websites, journal articles, and online support groups to quickly improve their food allergy health literacy [27]. This time-dependent need for finer assessments of credibility and expertise is something we do not feel has been clearly demonstrated in the literature. Nonetheless, Metzger and Flanagin’s [16] observations around receiver characteristics such as past experience, reliance, and prior knowledge are associated with this, but the focus here is more on experiences as a patient with food hypersensitivity as opposed to experience as a social media user per se.

Interview discussions demonstrated clearly defined groups of FH users on various social media platforms (eg, Twitter discussion participants or members of Facebook support groups) and supportive groups similar in nature to those recognized by Broome et al [27], Greene et al [1], and Hamshaw et al [30]. Groups supported fellow users when information or advice was needed, and drawing on and deferring to the knowledge of others (even when considered an expert yourself) was considered a highly regarded trait in someone supporting the community. A similar finding is presented by Lovatt et al [37], where use of caveats relating to one’s level of expertise was key to the development of trust in online breast cancer forums. Trusted familiar users (either traditional or experience experts) on social media were imbued with the ability to convey social validation such that their reactions to other users acted as a benchmark of status or believability. In a similar way to Metzger et al’s [29] findings around the use of social information pooling (such as reliance on testimonials, reviews, or ratings), social validation was conveyed here in FH users’ liking, sharing, or commenting on posted messages, which demonstrates a form of rating for the social media post itself. However, as suggested by the name, *social* media perceptions of credibility can involve a much more social assessment—users can partake in 2-way interactions, question authors of original content, and ask advice of other trusted users—thus, highlighting variance with typical observations relating to Web-based credibility assessments associated with sources that are more static. The credibility of expert knowledge was also visible within social media sources such as Twitter discussion groups such as #AllergyHour and Facebook support groups. Again, a factor that sets social media cues to expertise apart from those associated with typical Web sources was the level of engagement expected to validate expertise; for example, taking part in discussions, challenging misinformation, and being available to comment, was also noted as encouraging trust through social media by Lovatt et al [37]. This further highlights an affordance of social media and a different way that expertise can be assessed through the internet in a more hands-on fashion because of communication capabilities of these platforms.

When considering research around the more static forms of Web-based media such as websites and assessments of their credibility, findings may relate to social media, but the multidimensional nature of these platforms was not always seen to translate very easily. Frameworks relevant to assessments of Web-based information, such as those presented by Metzger and Flanagin [16] and Fogg et al [32], must now move further to account for the more complex nature of social media information. Users are assessing information that blurs the boundaries between source, message, and author—does one assess the post itself or the platform it resides on? Does the post come directly from the poster or has it been *shared* or quoted from elsewhere? Although our findings highlight many credibility cues suggested by frameworks, such as information recency, accuracy, and relevance, as well as author qualifications or credentials, and absence of commercial motives, it is clear that social media do not fit these molds well. Furthermore, platforms such as Twitter offer users regular real-time updates (through hashtags) on matters of interest, but because of the limited (although recently extended) character capacity for tweets, credibility assessments are more challenging. Social media posts often do not have the space to give as much detail as a website might to suggest expertise (eg, references, evidence, and associations with reputable organizations). Recent research has shown that links to other sources of evidence in social media posts can promote a sense of credibility [12]; however, the extent to which this can be considered the same as references or evidence cited within Web-based sources needs to be considered.

### Limitations

The interview sample was only a small number of social media users. However, it did consist of an array of FH concerns, from FH adults, parents of FH children, allergic and celiac, as well as those who make a living around food hypersensitivity (eg, writing about it or working for support organizations). However, the addition of 143 shorter yet detailed answers to questions relating to reasoning behind judgments of expertise for FH social media sources strongly enhanced the more in-depth interview sample. Several potential experts in the health care sector on social media were targeted during recruitment, and although 3 individuals did give informed consent, they did not respond during our interview timescale. Thus, we were not able to consider this perspective. Participants who had given informed consent and sent the first phase of interview questions were sent a reminder email if responses were not received within a reasonable time frame (approximately 1-2 weeks). Further reminders were not sent to avoid harassing participants who may have decided they no longer wished to take part in the email interview. Moreover, the gender split of the study sample could be considered imbalanced. Such an outcome has frequently been seen in the associated literature [5,55-57] and may be due to the more common primary caregiving role of females in managing a child’s food hypersensitivity. Unfortunately, the nature of sampling for a narrow population (individuals managing food hypersensitivity) limits the level of control over such considerations.

The email interview approach gave participants a high level of control over their data; they could consider replies, gather information, and add to previous responses. This reflection time slows down the research process, and the lack of face-to-face contact means participants can more easily ignore or forget about questions. Reminders proved useful in some cases, but it was difficult to know when to start and stop prompting. Compared with face-to-face interviews, developing rapport with participants was more challenging because of lack of social cues. Participants had their own communication styles, and we needed to adapt to these. Creating an interview schedule also presented additional issues. There is little opportunity to prompt participants, and confusing questions might lead to withdrawal. It was essential that questions were clear and likely to promote rich detailed responses. We also encouraged participants to be as detailed in their responses as possible. Thus, email and face-to-face interviews need to be viewed as distinct research approaches, each requiring a slightly different set of skills [58].

### Conclusions

This study has begun to unpick factors associated with constructions of expertise on social media, specifically in the area of food hypersensitivity. Traditional perceptions of expertise, such as formal qualifications, remain a taken-for-granted sign of expertise; however, it was acknowledged that those living with food hypersensitivity could be seen as experts through their lived experience. There appear to be several cues to FH expertise on social media, including those typically anticipated such as factual and appropriate information and evidence. The 2-directional (*social*) nature of social media highlighted how social validation cues, such as likes, shares, follows, comments, and communication with other reputable sources or users, could aid in the assessments of expertise in a different way to more static forms of Web-based media.

Future work would benefit from exploring constructions around expertise on social media from the perspective of those considered traditional experts and how experiential expertise is considered here. This study suggests that more support may be needed in relation to living with food hypersensitivity, especially following diagnosis. Exploring approaches that encourage the mutual support of traditional and experienced patients and carers in managing health concerns (eg, online) could prove valuable. Understanding the processes involved in social media information assessments could help support groups to design interventions to improve the information evaluation skills of social media users; such applications could prove vital, as people increasingly turn to Web-based sources for help and support in relation to their health. Practical and actionable implications from the study findings may include the following:

1. Providing further support for those with food hypersensitivity following diagnosis. This may be through additional and subsequent contact with their medical diagnostic team.
2. Instigate online platforms that could foster mutual support from medical professionals and those who have experience managing food hypersensitivity on a day-to-day basis, for instance, more tailored forums or chat rooms, which could be closed to the public and moderated.
3. Develop the provision of training for medical professions in use of social media. For example, in how to promote one’s own expertise but also manage impressions given to the expertise of other users on social media (eg, through their own practices or that of other users). A stronger understanding of these issues would also enable practitioners to empower their patients in managing such concerns.
4. Stakeholders not only need to consider the accuracy of the information they post on social media but also the degree they evidence their posts. In addition, relevance was seen as a key issue here, meaning stakeholders may need to consider how they tailor their communications to target certain audiences.

## Appendix

Multimedia Appendix 1 Email interview schedules.

## Abbreviations

FH: food hypersensitive
MS: multiple sclerosis
