# Supplementary material for: Perceptions of Food Hypersensitivity Expertise on Social Media: Qualitative Study
Source: Interact J Med Res. 2019 Jun 28;8(2):e10812. doi: 10.2196/10812 (PMC6625221; doi:10.2196/10812)
Supplement: Multimedia Appendix 1 [file ijmr_v8i2e10812_app1.pdf]

Appendix

Email Interview Schedules

1. FH Participants

Phase 1 Questions

Q1 Can you tell me a bit about the way you typically use social media for things that relate to your food allergy or intolerance?

Perhaps you have some specific examples of things you tend to do, or accounts or people you tend to follow or share information with?

You might like to attach image examples (e.g., using the Windows snipping tool) if it helps.

(Please be reassured that information about others will be kept anonymous)

Q2 What do you think it is about a user or account relating to food allergy or intolerance on social media that leads you to consider them as an expert?

Please say as much as you can about this – and please do give examples if you can. Of course, there are no right or wrong answers to this – we are genuinely interested in your views about expertise.

Q3 You mentioned ____________________* as a potential expert on social media in relation to food allergy during our online survey study. Can you talk me through your reasoning behind giving ________________* as an example? What do you think it is that this person does that leads you to consider them as a potential expert?

Example Phase 2 Questions

Q4 Last time we spoke about some of the reasons why you considered _____________________* an expert on social media around food allergy/intolerance.

I would be interested to hear how you think ____________________* might have come to be seen as an expert by others.

Q5 Do you feel that how we judge the expertise of others on social media is different to how we might judge expertise in other settings (for example, offline, or elsewhere on the internet)?

Please could you give some detail about your thoughts?

Perhaps you could relate your thoughts to the area of food allergy/intolerance?

Q6 Finally, it would be really interesting to hear some of the ways you might consider something or someone on social media as non-expert or untrustworthy?

Do you notice specific things, or do you have any examples?

** include reference to the expert(s) participants cited in their survey response.*

2. Perceived Experts

Phase 1 Questions

Q1 Can you tell me a bit about the way you typically use social media for things that relate to food allergy or intolerance?

Q2 What sorts of things do you think are important when others judge the expertise of another social media user?

How about specifically in relation to food allergy and intolerance?

Are there perhaps any social media users that you feel are expert in this area?

Please say as much as you can about this – and please do give examples if you can. Of course there are no right or wrong answers to this – we are interested in your views about expertise.

Q3 As you know, some food allergic/intolerant social media users in a survey we recently conducted identified you as an expert in food allergy/intolerance on social media – what do you think people's reasoning might be for this judgement?

Were you surprised to hear you were mentioned by others?

Example Phase 2 Questions

Q4 Can you talk me through the sorts of things you consider before posting something on social media around the topic of food allergy/intolerance? For example, do you consider where your information has come from, who might read it, whether you should use things like hashtags or mention other social media users etc.?

Can you tell me about a typical exchange during your food allergy/intolerance social media activities?

Q5 Do you think social media has changed the way you think about yourself, for example as someone with expertise in this area?

Q6 Do you feel that how we judge the expertise of others on social media is different to how we might judge expertise in other settings (for example, offline, or elsewhere on the internet)?

Please could you give some detail about your thoughts?

Perhaps you could relate your thoughts to the area of food allergy/intolerance?

Q7 Finally, it would be really interesting to hear some of the ways you might consider something or someone on social media as non-expert or untrustworthy?

Do you notice specific things, or do you have any example?
